# A mixed methods descriptive investigation of readiness to change in rural hospitals participating in a tele-critical care intervention

**DOI:** 10.1186/1472-6963-13-33

**Published:** 2013-01-29

**Authors:** Jane Zapka, Kit Simpson, Lara Hiott, Laura Langston, Samir Fakhry, Dee Ford

**Affiliations:** 1 Department of Public Health Sciences, Medical University of South Carolina (MUSC), 135 Cannon Street, Charleston, SC, 29425, USA; 2 Department of Health Leadership and Management, MUSC, 151 Rutledge Avenue, Charleston, SC, 29425, USA; 3 Division of Pulmonary, Critical Care, Allergy, and Sleep Medicine, MUSC, 96 Jonathan Lucas Street, Charleston, SC, 29425, USA; 4 Department of Surgery, MUSC, 96 Jonathan Lucas Street, Charleston, SC, 29425, USA

**Keywords:** Tele-medicine, Rural hospitals, Readiness to change

## Abstract

**Background:**

Telemedicine technology can improve care to patients in rural and medically underserved communities yet adoption has been slow. The objective of this study was to study organizational readiness to participate in an academic-community hospital partnership including clinician education and telemedicine outreach focused on sepsis and trauma care in underserved, rural hospitals.

**Methods:**

This is a multi-method, observational case study. Participants included staff from 4 participating rural South Carolina hospitals. Using a readiness-for-change model, we evaluated 5 general domains and the related factors or topics of organizational context via key informant interviews (n=23) with hospital leadership and staff, compared these to data from hospital staff surveys (n=86) and triangulated data with investigators’ observational reports. Survey items were grouped into 4 categories (based on content and fit with conceptual model) and scored, allowing regression analyses for inferential comparisons to assess factors related to receptivity toward the telemedicine innovation.

**Results:**

General agreement existed on the need for the intervention and feasibility of implementation. Previous experience with a telemedicine program appeared pivotal to enthusiasm. Perception of need, task demands and resource need explained nearly 50% of variation in receptivity. Little correlation emerged with hospital or ED leadership culture and support. However qualitative data and investigator observations about communication and differing support among disciplines and between staff and leadership could be important to actual implementation.

**Conclusions:**

A mixed methods approach proved useful in assessing organizational readiness for change in small organizations. Further research on variable operational definitions, potential influential factors, appropriate and feasible methods and valid instruments for such research are needed.

## Background

Broadly considered, telemedicine is a tool linking clinicians and patients otherwise separated by distance. It has intuitive appeal in settings where a clinical need cannot be optimally met by local clinicians and could theoretically lessen health care disparities experienced in rural communities. Through the use of telemedicine, physicians in multiple specialties have been able to provide cost effective care to rural and underserved patients [1-4]. Telemedicine alleviates some of the issues rural physicians experience, including lack of onsite specialists, isolation, poor communication, and lack of access to current medical information and continuing medical education [2]. Patients’ perception of the quality of care may be improved via telemedicine [5] and financial impact of transfer avoidance may be reduced [6]. Despite these demonstrated and potential benefits, the literature reveals that hospitals and rural physicians have been slow to adopt telemedicine initiatives [7-9].

Dissemination and diffusion efforts have been increasingly highlighted as a priority to improving population health and reducing disparities [10-13]. Yet numerous challenges for adoption and implementation at the provider and organizational levels exist and many are poorly understood. Thus, a better understanding of the factors in the contextual domains which impact decisions to adopt new interventions is needed [10,14,15]. Related to this concern are theories of readiness to change and implementation [9,10,16,17] and a need for rigorous assessment tools [18-20].

Given these priorities, this paper profiles the contextual factors associated with readiness to participate in an innovative telemedicine project designed to improve care for patients presenting at rural hospitals with high acuity conditions – sepsis and trauma. We investigated key elements of the provider and organizational context as reported by hospital leadership, explored relationships of these data with hospital staff survey ratings and triangulated these data with the observational reports of investigators.

## Methods

### Setting and intervention

South Carolina (SC) is a state in which rural communities disproportionately experience health care disparities [21]. Given severe resource limitations and a national shortage of critical care clinicians [22,23], rural SC communities are unlikely to obtain critical care experts on site who can optimally manage severe sepsis and trauma. Thus, with external funding (NIH/NIMHD, RC1MD004405-01) the Critical Care Excellence in Sepsis and Trauma program (CREST) was established at the Medical University of South Carolina (MUSC). The overall objective of CREST is to improve the timeliness and quality of key treatment decisions in sepsis and trauma. To accomplish this, CREST partnered with four rural SC hospitals’ Emergency Departments to disseminate clinician education and telemedicine consultation services and strategies. CREST hospitals were chosen from a geographic region of SC along the Interstate-95 corridor which transects the state. This region of SC has marked health disparities including increased mortality from sepsis and trauma and a high proportion of minorities and citizens living below the federal poverty level. Hospitals were then selected for participation based on two criteria: designated as rural by the US Department of Agriculture and designated as fully medically underserved [21]. The sites have no formal affiliation agreement with MUSC. Together the 4 hospitals serve 165,500 citizens with 27% of the population living below the federal poverty level [24].

CREST was implemented in a stepwise fashion at partner hospitals in the following manner. First, administrative and regulatory issues were considered. This included memoranda of agreement (MOA), human subjects designation and IRB process, physician credentialing and privileging and explication of current and future financial obligations. Next an onsite clinical educational program in sepsis and trauma was conducted. The education included training in the use of the web-based telemedicine equipment. The final phase was activation and utilization of the telemedicine consultation service which was embedded into existing MUSC clinician on-call systems. During the grant period no reimbursement to the lead organization was required as it was covered by grant funds. CREST remains ongoing.

### Design

This is a multi-method, observational case study. There has been increasing research interest in mixed methods research as it provides a more comprehensive picture of health services than individual methods alone [25,26]. We employed a core qualitative component as well as a supplementary quantitative component [27]. Data were collected during the implementation and medical education phases of the CREST intervention in 2010–2011. This study was approved by our Institutional Review Board which is responsible for overseeing the ethical conduct of human subjects research. Key informants gave signed informed consent prior to participation and a waiver of signed consent was granted for survey respondents.

### Guiding framework

**Figure 1:**
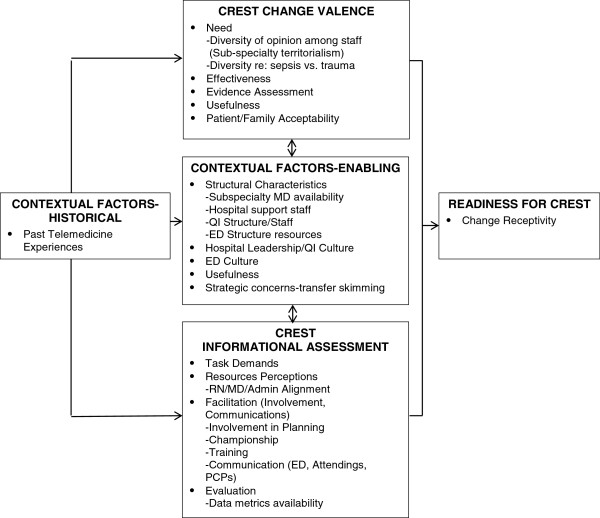
**Determinants and outcomes of organizational readiness to adapt CREST.** Adapted from Weiner [28], Helfrich [18], Hu [9], Stetler [29].

Conceptual frameworks which integrate the theoretical domains of multiple disciplines are receiving increasing attention given convincing evidence of the need to understand the context in which change and adoption take place [14,20,28,29]. In this study we adapted elements of an organizational readiness for change model proposed by Weiner [28]. We incorporated factors and items suggested by Stetler [29] and Helfrich and colleagues [18]. These included such factors as clinician perceptions about the evidence related to efficacy of telemedicine, and factors related to facilitation of adoption such as local champions and available resources. Other investigators have discussed related factors consonant with a broad readiness model [20]. For example, the Content, Context and Process Model of the strategic management of change [30] urges differentiation between non-receptive and receptive features of the organizational context. Hu and colleagues [9] purport that behavior potential is predicted by attitudes, perceived usefulness and perceived ease of use. In our observations and inquiries, we were also guided by the classic domains which affect quality of care, factors related to structure, process and outcome [31]. Alexander and Hearld [32] emphasize the need to study multiple perspectives on delivery system design and Weiner [28] urges that readiness items should focus on the specific impending change which we did in our data collection queries (i.e., reference to CREST). Broadly, our model included readiness for CREST, the dependent measure in this paper; and four potential mediating domains; *historical contextual factors* such as experience with host institution and other telemedicine projects; e*nabling generic contextual factors* of the organization such as leadership, staff structure; and two CREST specific domains, *“change valance” factors* which could negatively or positively impact decisions to participate, such as perception of need, and awareness of scientific evidence and *assessment of CREST characteristics*, such as task demands and resources needed see Figure 1.

### Data sources and collection

Three data sources were used: systematic project field notes, key informant interviews with CREST hospital leaders and selected staff, and a self-administered survey of clinicians at the four participating hospitals.

As CREST planning and training proceeded, *systematic field notes* were kept by investigators. During contacts via telephone, email, and in-person meetings, we noted observations about dynamics. Investigators’ observations were broadly organized as “collaboration dynamics” (MOA, completion of certifications related to human subjects research, credentialing and privileging) and “training dynamics” (planning, scheduling). Investigators summarized these observations and impressions according to the broad domains of the readiness model.

*Key informant interviews (KIIs)* were conducted to glean motivations and concerns of clinicians and administrators. KIIs were held primarily in person at the hospital, but occasionally by telephone because of difficult scheduling. Interviews lasted from 20–40 minutes, recorded with permission, and with back-up hand notes. We asked open ended items about perceptions of evidence, need, the hospital and Emergency Department context and facilitation elements [33].

The CREST *clinician survey* items were informed by the literature, investigators’ observations and KII data. Mindful of respondent burden, we adopted or adapted parsimonious dimensions of domains suggested by organization theory [28]. Items queried participants about perceptions, experiences and ratings, with a 6 response category choices ranging from 1–6 from “strongly agree” to “strongly disagree”. A choice of “don’t know/not applicable” was provided. Within the *Hospital Context* domain, three factors were queried: leadership and quality improvement (5 items), ED culture (5 items) and strategic concerns such as hospital transfers (2 items). Within the *CREST Change Valance* domain, defined as how staff value a specific impending change [28], three factors were investigated: evidence assessment (1 item), need (3 items), and usefulness (3 items). Within the *CREST Information Assessment* domain, 4 factors were queried: task demands (5 items), resource availability (3 items), involvement/communication (3 items), and evaluation needs (4 items). Change readiness was measured by one item “ED staff is receptive to CREST”. The CREST survey was self-administered anonymously at the start of the CREST educational session at each hospital.

### Analytic approach

Data from each source were first summarized for each hospital site; then responses were compared across sites. Qualitative data from the managerial reports and KIIs were sorted for themes related to the core domains and factors by two investigators (LH, JZ) and then reviewed by another investigator (DF) until consensus was achieved.

Given the small number of respondents, survey data were pooled across the hospitals. We reviewed the frequencies of responses to the individual items as we reviewed comments from the other two data sources [26]. Additionally, the 34 survey items were combined based on the content face validity and where they fit into the conceptual model domains. See Table 1 for the domains, factors and related items. This resulted in three summary scores where low scores indicated general agreement and high scores expressed disagreement. This was a crude method for exploring the extent to which survey responses agree with the hypothesized relationships posed by the innovation model. We used simple summation across responses and assigned a zero value to missing and not applicable responses. Thus, all factors had a possible low value of 0, but the high values varied. For example, a factor with three relevant items will have a maximum score of 18, while a factor with five items will have a possible score of 30. This process allowed the use of regression analyses to examine correlations between each factor and our proxy measure for commitment to CREST (ED staff are receptive to CREST). In the regression modeling, the Likert scale response to the dependent measure was used as a continuous variable. SAS 9.2 was used for survey data analyses [34].

**Table 1:** Survey domains, factors, and items

**CREST CHANGE VALANCE**	
**Need**	
	The need for CREST is supported by your clinical experience here at your hospital
	The need for CREST-Sepsis is supported by your clinical experience at your hospital
	The need for CREST-Trauma is supported by your clinical experience at your hospital
**Effectiveness/ usefulness**	
	Using CREST will enable me to complete patient care more quickly
	Using CREST will improve my productivity in patient care
	Using CREST will enable me to provide better quality care
**Evidence Assessment**	
	The CREST program strategy is strongly supported by key evidence in the clinical literature
**CONTEXTUAL FACTORS - ENABLING**	
**Hospital leadership/ QI culture**	
	Senior leadership/clinical management in your organization promote team building to solve clinical care problems at your hospital
	This hospital is formal and structured place. Bureaucratic procedures govern what people do
	Managers in this hospital are risk-takers. They encourage employees to take risks and be innovative
	Managers in this hospital are coordinators and coaches. They help employees meet the hospital’s goals
	Administration, staff, and physicians work together to improve patient care
**ED culture**	
	ED staff have a sense of personal responsibility for improving patients and outcomes
	ED staff (MDs, RNs and others) cooperate and improve effectiveness of patient care
	ED staff are willing to try new approaches to improve clinical procedures
	ED leaders work cooperatively with hospital leadership to make needed and appropriate changes
	ED leadership provides effective management for continuous improvement of patient care
**Strategic concerns-transfer, skimming**	
	There is a disagreement between different hospital groups on the desirability of keeping a patient versus transferring the patient
	How often is there disagreement between different hospital groups on the desirability of keeping a patient versus transferring the patient
**CREST INFORMATION ASSESSMENT**	
**Tasks demands**	
	Implementation of CREST is feasible at our hospital
	I am clear about my roles and responsibilities in CREST
	Learning to operate CREST technology will be easy for me
	Learning to operate CREST technology will NOT be easy for me
	The CREST project will require team work among all ED staff
**Resource perceptions**	
	There are enough MDs in the ED to implement CREST
	There are enough RNs in the ED to implement CREST
	We have the resources to implement CREST effectively
**Facilitation (involvement, communication)**	
	There is a clear leader or champion for CREST at our hospital
	Communication with staff about CREST planning has been excellent
	I was appropriately involved in the planning for CREST
**Evaluation**	
	It is important to collect feedback from patients regarding CREST
	Collecting feedback from staff regarding implementation of CREST will be important
	Developing and distributing regular CREST-related performance measures to clinical staff will be important
	It will be important to provide a forum for presentation/discussion of CREST’s progress and implications for continued improvement

Findings from all data sources were then triangulated or integrated [35]. While there are several operational definitions of triangulation [27], we use it here to provide rich data on potential content validity—that is reports of factors related to adoption. Additionally, we compare data from more than one source to confirm if an observation from one data source is evident in another data source [27]. In this exploratory research, we do not view any of the data sources as the “gold standard”, that is, we are not assessing criterion validity.

## Results

Selected organizational characteristics of the participating hospitals are reported in Table 2. Significant proportions of the hospital service area population were below the federal poverty level and are minorities; only one hospital had trauma level status.

**Table 2:** Participating hospital characteristics

	**Critical access hospital**	**% County population that is minority (range)**	**% County below federal poverty level (range)**	**Joint commission accredited**	**# of Beds (range)**	**Trauma level**
**HOSPITAL A**	No	40-49	20-29	Yes	50-99	N/A
**HOSPITAL B**	Yes	60-69	30-39	Yes	< 50	N/A
**HOSPITAL C**	No	60-69	30-39	No	50-99	N/A
**HOSPITAL D**	No	60-69	20-29	Yes	200-350	Level 3

*All sites are Rural County, Federal #4, in medically underserved areas and are Medical Facility Status Level 3.

A total of 23 KIIs were conducted with 6, 5, 4, and 8 interviewees respectively at hospitals A-D. Interviewees included Emergency Department Medical and Nursing Directors, physicians (e.g. ED, hospitalists), Quality Improvement Managers/leaders, Chiefs of Staff and Nursing, and Chief Administrators.

There were 86 completed staff surveys from the 4 hospitals with 13, 15, 2 and 56 surveys respectively at hospitals A-D. It is difficult to provide an accurate response rate of eligible participants, given the variable and frequently changing number of part-time and rotating staff on shifts in very small resource poor hospitals. Respondents included physicians, nurses, EMS personnel, and a sampling of other disciplines. Findings are reported by domain and factor (Figure 1) and by data source.

### Historical contextual factors

KIIs from two hospitals reported experience with telemedicine programs, notably an MUSC tele-stroke program (REACH) [36] and a SC Department of Mental Health (SC DMH) tele-psychiatry program [37]. It was evident that this experience enabled support for CREST. One KII commented that “REACH is the word. Everyone wants CREST because of the success of REACH”. Another noted that the quality of patient care had increased – length of stay, choice of medications, communication between ED physicians and psychiatrist, patient satisfaction—because of SC DMH Tele-psychiatry.

With respect to previous interaction with MUSC, some concern was expressed by interviewees from 3 hospitals about the importance of getting feedback communication on the patients transferred to MUSC. Two hospitals stressed the need to communicate processes of care with local primary care physicians. Distance to MUSC was mentioned several times. One asked if MUSC would resist accepting transfers of indigent patients.

### CREST Change valance

KII and staff survey data both reflected important positive and negative factors which could affect readiness to be involved with CREST.

There was general agreement among KIIs at all hospitals on the need for CREST. Responses highlighted need because of lack of specialty staff and resources. Between hospitals however, there were differences of opinion on the consultation need for sepsis as compared with trauma. For example, KIIs from one hospital with a very large geriatric population emphasized their need for help with sepsis cases. Another felt great need for assistance with trauma cases as neurosurgical and orthopedic specialists were not available. In three hospitals, there was difference in KII rating of need by discipline—since ED physician staff was contracted and often changing, the nursing staff emphasized the need for CREST more so then did the physicians. The perceived need was corroborated by the survey item “the need for CREST is supported by your clinical experience here at your hospital” to which 78% responded they strongly agreed and another 13% moderately agreed (mean=1.36).

In the KIIs, there was next to no familiarity with or mention of the scientific evidence related to telemedicine efficacy. However 65% (mean=1.56) of survey respondents reported they strongly agreed that “the CREST program strategy is strongly supported by key evidence in the clinical literature”. KII comments indicated a disposition toward “common sense” observation that a resource for expertise was necessary and would be effective. With respect to “evidence”, interviewees at two hospitals again emphasized their experience with the REACH and SC DMH Tele-psychiatry programs.

When reflecting on potential usefulness of CREST, interviewees from all hospitals commented on the potential usefulness of CREST to improve the quality of care for patients, “quicker” consults and better outcomes. When queried about patient/family acceptance, all KIIs felt that the response would be very favorable: “patients will be amazed”; “it will be seen as a blessing.” Survey respondents reported that CREST would enable them to complete patient care more quickly, would improve productivity and enable them to improve quality care 42% (mean=2.05), 45% (mean = 1.68), and 52% (mean=1.99), respectively, strongly agreed.

### Enabling contextual factors

The structural features of the hospital’s organization were highlighted by KIIs as well as by CREST investigators’ notes. Most frequent were comments on availability of specialist staff. In one hospital, administrative issues were difficult with leadership changing three times among contracted management firms and because of poor physical plant for administration and the ED itself. Interviewees at several hospitals pointed out that the ED physicians, including the Medical Director were not hospital employees, but rather contracted physicians and frequently were hard to reach and/or had limited hours.

With respect to leadership and the structure and processes of their quality improvement activities, different features were highlighted. High-level leadership was at least nominally supportive, and was highly present in two of the hospitals according to both KIIs and project management notes. The QI presence was quite variable. At only one hospital was a structured QI unit championing CREST and coordinating the planning and implementation process. The responses to survey items related to hospital leadership culture (not necessarily focused on CREST) demonstrated less agreement that “hospital leadership encouraged employees to take risks and be innovative” (9% strongly agreed) or promoted team building to solve clinical care problems at the hospital (23% strongly agreed). In fact the survey items about hospital leadership quality had higher means, i.e. stronger disagreement, than all the other groups of items.

The observations in the staff field notes about communication processes were noticeably different among the hospitals. For example at one hospital, the medical staff and the ED staff appeared in regular communication, not so at other hospitals. The ED culture at two hospitals appeared as functional and organized—as illustrated by survey comments about nurse-physician collaboration -- the nursing staff as “willing to learn” and all clinicians as “responsive to change”. Championship by ED directors varied from “being distant” to being committed to communication and sharing. Two camps were evident in KII statements at one site, “enthusiastic young Turks and the old guard”. Survey items referring to the ED culture were rated generally in agreement but not strong agreement. For example, approximately 25% of respondents strongly agreed that “ED staff (MDs, RNs and other) cooperate and improve effectiveness of patient care”, and “ED staff have a sense of personal responsibility for improving patients care and outcomes”. The KIIs and investigator observations however frequently highlighted the importance of nursing staff leadership in day to day functioning.

Several strategic concerns were evidenced in the KII reports and also in the interactions with CREST investigators. Concern about “patient stealing” was heard from only one hospital. More common were the remarks about CREST helping with hospital survival (two hospitals), improving public confidence and trust and improving statistics with state regulators.

### CREST Informational assessment

Interviewees offered many comments on CREST task demands and resource availability. A KII from one hospital stressed that the technology would have to be easy to use. One wondered if the tasks would result in increased time to disposition. Participants from all hospitals stressed that the education/training would be very important; “…training will make it or break it as there are many part-timers who are at some distance away”. All interviewees from one hospital in particular commented on the lack of space in the ED. KIIs at one hospital discussed their close collaboration with the local area EMT service as a strength in its collaborative network.

Several survey items queried about CREST resource assessments. Sixty one percent (mean=1.62) strongly agreed that implementation was feasible at their hospital, but only 32% (mean=2.32) felt their roles/responsibilities were clear; 48% (mean = 1.81) strongly agreed CREST would require ED staff teamwork. About one-third of respondents strongly agreed there were enough resources and enough nurses to implement CREST (2.32 and 2.18 respectively); 40% (mean = 1.97) strongly agreed there were enough MDs for implementation.

KIIs were asked about their involvement and communication among personnel to date. Response varied but most felt they were at least somewhat informed. At two of the hospitals, KIIs referred to particular “champions”. In two hospitals, comments about the challenges of communication were voiced by CEO/senior administrator and the nurses as there was no real ownership at the ED level. In the survey, while 36% (mean= 2.12) of respondents strongly agreed communication with staff had been excellent, 27% (mean=2.85) strongly agreed they had been appropriately involved. The reality of facilitating the implementation of CREST was most evident in the structured notes of the staff and investigators. Managerial notes chronicled the interactive processes of developing and signing a MOA, applying for Federal Wide Assurance as required in human subjects research, and establishing credentialing and privileges. The time and energy needed by CREST staff was judged to be directly related to the organization's available support staff and efficiency. Time for completion of administrative tasks ranged from 11–12 months, and would have actually been longer in one or two without diligent administrative prodding.

With respect to evaluation expectations and availability of metrics for success, several KII comments were offered. Most common was the mortality rate pre- and post- CREST implementation, particularly for sepsis. Getting feedback on transferred patients from MUSC was again mentioned. In the survey, more than half strongly agreed that collecting feedback from staff, developing and distributing CREST performance measures to staff and providing a forum for discussion of implications for continued improvement.

### Readiness for CREST and related domains and factors

The survey responses to the item “ED staff is receptive to CREST” were 42% strongly agree, 23% moderately agreed, 32% agreed, 2% disagreed and 1% moderately disagreed (n=20 missing).

Table 3 reports the exploratory findings from the regression model of independent associations of the summary measures with the rating of receptivity to CREST. The results of the five-factor model (Model 1) show that the factors were not equal in explanatory power. The summary measure of perceived need for CREST is the most important factor in explaining the variations in CREST receptivity. The summary measure of hospital leadership and QI culture had the lowest explanatory power and removal of this factor from the model improved explanatory power. The factors that measure aspects of ED culture also contributed little to explaining variation. This factor contains items related to cooperation between hospital administration and ED leaders to make needed changes, ED staff responsibility for care quality, cooperation between ED staff, and willingness of ED staff to try new approaches.

**Table 3:** Regression models and factors measures associated with level of CREST receptivity

**Summary measure**	**Model 1 Beta (p-value) N=60**	**Model 2 Beta (p-value) N=60**	**Model 3 Beta (p-value)**
Task Demands	0.06787	0.06751	0.07358
	(0.0772)	(0.0728)	(0.0248)
Resource Perceptions	0.09839	0.09800	0.11661
	(0.0209)	(0.0189)	(0.0015)
ED Culture	0.02817	0.02726	-
	(.2909)	(0.2214)	
Need for CREST	0.46691	0.46907	0.45350
	(0.0156)	(0.0128)	(0.0060)
Hospital QI Culture	−0.00153	-	-
	(0.9488)		
Intercept	−0.9025	−0.09956	0.6886
	(.81)	(0.7712)	(0.7962)
R^2^	0.4852	0.4851	0.5111
Adjusted R^2^	0.4375	0.4477	0.4878
	(model <0.0001)	(model <0.0001)	(model <0.0001)

The three factors that explain nearly 50 percent of the variation in responses to the change commitment measure are “need for CREST”, CREST “task demands” and perceived “resource needs” relevant to implementation. Respondents who strongly agreed to the “task demands” items also strongly agreed that ED staff is receptive to CREST. Respondents who strongly agreed that CREST is feasible, the technology is easy to use, their roles vis-à-vis CREST are well defined and ED teamwork is required for CREST to succeed, also recorded a strong CREST commitment score. Individuals who strongly agreed to the “resource perception” questions (we have enough physicians and nurses and general resources to implement CREST) also reported strong CREST receptivity scores.

## Discussion

The domains and factors that support or hinder the implementation of change in the hospital care processes is an important area of research, and our study results demonstrate the importance of using a mixed-methods approach to examine them. The use of this approach may be especially important for research focused on issues in small organizations with their inherent sample size limitations and variable and changing structural and communication characteristics. Our study findings support important aspects of the conceptual model of adoption of innovations, yet support the call for intensive work on instrument development for this area of inquiry [32] and need for intensive multi-variate and time analyses [17,32].

From a managerial or implementation perspective, the findings reveal that in order to foster individual staff intentions to adopt and use a technology, the perceived clinical need and usefulness is crucial. Clarification of, and communication about, task demands is also key. While the findings from the regression models were exploratory, they indicate that individuals who strongly agreed there was need for CREST, resources for implementation were adequate, CREST technology was easy to use and that their personal role was well defined were most likely to agree that there was high commitment to CREST. Interestingly in our study, the summary measure “hospital leadership and quality improvement culture” contained five questions related to hospital-wide perceptions of team building, organizational structure, perceived management attitude toward risk taking, and management and medical staff working together to improve quality of care and these were less related to CREST commitment. This finding is somewhat counter to recommendations from general change management literature, that it is essential to have management commitment and the support of a local champion [38,39]. Perhaps the participating organizations were characterized by decentralization, with broadly defined jobs, making hierarchical leadership less of a factor. It may be more important to assure that we “sell the idea” to the individuals who provide the actual care when we implement changes in small institutions. This issue requires further investigation.

Previous rigorous work has applied numerous models and highlights the challenges of theory-testing and developing more valid measurement tools. For example, these include the Content, Context Process Model [29], the Technology Acceptance Model (TAM) [9], the Promotion Action and Research Implementation in Health Services Model (PARIHS) [18] and the Evidence, Context and Facilitation Model [33]. All investigators championed the need for theory testing and validated measures. Published reviews indicate that to date instruments for measuring organizational readiness for change demonstrate variable operational definitions and little evidence of reliability or validity [17,32,40]. Conceptually, many of the domains and factors discussed in various models are similar. The challenge is to establish generalizable construct and content validity within survey instruments. Clearly this is not an easy task, given health care organizations are complex adaptive systems, [41,42] as are physician-organizational relationships [43].

Indeed, much needs to be done. Meanwhile the continued application of mixed methods is urged to promote triangulation of findings and prompt debate [27,32,44]. The qualitative comments and observations in the CREST study for example elicited undercurrent comments about need for feedback from MUSC to local providers and primary care clinicians. Effective communication between hospitals will likely increase in importance in the context of the Patient Protection and Affordable Care Act that includes a mandate to develop patient-centered medical homes [45].

Clearly more qualitative observation of variability will inform item construction choices for quantitative measures. Kitson and colleagues [33] remind us of the need to be mindful that while clinical effectiveness agenda stress the level and rigor of evidence as the most important factor for consideration, other factors may be critical. Review of the CREST management notes, reminded CREST investigators to be cognizant of the method or way the process is facilitated. For example, the factors of facilitation [33] consider the characteristics of the facilitators, their role and their styles. These facilitation factors not only apply to the champions at respective sites, but to the scientific leadership at academic leadership sites. These should be the target of more exploratory research. Jean and colleagues illustrate that while a multi-method approach is challenging it is feasible and vital to understanding the process and outcome of an innovation implementation process [46]. Work in other areas such as cancer, has highlighted that care processes have numerous task and interfaces, all of which can impact the ultimate implementation of quality care [47]. Implementation of innovation will require flexible and variable processes. Additionally, an intervention may well require several multilevel strategies which must be carefully considered in a causal framework [48,49].

As projects and collaborations develop across the U.S., interest and readiness for telemedicine interventions will undoubtedly increase. Notably, the collaboration/negotiations with the two CREST hospitals with experience with REACH and SC DMH Tele-psychiatry were more enthusiastic and efficient. This trend should also be reinforced by the overall increase in use of web-based media by physicians as well as the general public [50]. This secular trend, a potential threat to internal validity, will however provide challenges to the design of studies aimed at establishing efficacy and effectiveness of innovations.

We acknowledge several limitations to this work. Clearly we have a small sample size at both the organization and individual clinician levels, which limit generalizability and cross-hospital comparisons. For example, Weiner [28] emphasizes that within the change valance domain, perceived value may not be exhibited consistently in cross-site relationships, even if readiness to change measures are similar. We were not able to obtain details of the numbers of full-time and part-time staff on all shifts at all hospitals and thus are unable to determine a response rate. This calls for caution in interpreting results because of selection bias. Also given the survey was administered at the continuing education program itself, our results reflect selection bias. As with any survey, the potential effects of social desirability in responses is a threat to internal validity which must be considered. For example while it was clear from the qualitative data that staff were unaware of empirical evidence, 65% of survey respondents strongly agreed the evidence supported the CREST intervention. Our sample size was too limited to report rigorous factor analyses on the survey items. As discussed above, vigorous psychometric work on survey and scale development must be a high priority.

## Conclusions

It is clear that the use of an exploratory mixed methods approach is valuable in the examination of domains and factors affecting technology adoption and implementation in small organizations. The importance of staff perception of both a clinical need and usefulness of an intervention may be crucial factors for successful implementation. Clarification of, and communication about task demands associated with the change is also a key variable. Factors related to top management support and leadership may be less important in these smaller organizations, perhaps because of frequent changes due to contract management, or due to differences in structures and job responsibilities in small organizations. Importantly, our study design and findings indicate that there is a great need for further research on instruments to measure the domains and factors related to implementation of evidence based interventions. Studies should assess important operational definitions which reflect the developing models and theories about adoption and institutionalization of change in health systems of variable size and structure.

## Abbreviations

CREST: Critical Care Excellence in Sepsis and Trauma; MUSC: Medical University of South Carolina; MOA: Memorandum of Agreement; KII: Key Informant Interview; QI: Quality Improvement; ED: Emergency Department; REACH: Remote Evaluation of Acute Ischemic Stroke; SC DMH: South Carolina Department of Mental Health; CEO: Chief Executive Officer.

## Competing interests

The authors declare that they have no competing interests.

## Authors’ contributions

JZ was leader of the conceptualization and design of the study, data collection, analysis and interpretation of project management, qualitative and quantitative data, drafting and revising the manuscript. KS contributed to the conceptualization and design of the study, conducted analyses and reporting of the quantitative survey data, reviewed qualitative findings and revision of the manuscript. LH participated in the analysis of the qualitative data and the drafting and revision of the manuscript. LL participated in the data collection and organization of project management reports and the revisions of manuscript drafts. SF contributed to the review of documentation and organization of the project management reports, review and modification of the manuscript draft. DF contributed to the conceptualization and design of the study and the analyses and interpretation of managerial, qualitative and quantitative data, and the drafting and revising of the manuscript. All authors read and approved the final manuscript.

## Pre-publication history

The pre-publication history for this paper can be accessed here:

http://www.biomedcentral.com/1472-6963/13/33/prepub
